# Gene-by-Psychosocial Factor Interactions Influence Diastolic Blood Pressure in European and African Ancestry Populations: Meta-Analysis of Four Cohort Studies

**DOI:** 10.3390/ijerph14121596

**Published:** 2017-12-18

**Authors:** Jennifer A. Smith, Wei Zhao, Kalyn Yasutake, Carmella August, Scott M. Ratliff, Jessica D. Faul, Eric Boerwinkle, Aravinda Chakravarti, Ana V. Diez Roux, Yan Gao, Michael E. Griswold, Gerardo Heiss, Sharon L. R. Kardia, Alanna C. Morrison, Solomon K. Musani, Stanford Mwasongwe, Kari E. North, Kathryn M. Rose, Mario Sims, Yan V. Sun, David R. Weir, Belinda L. Needham

**Affiliations:** 1 Department of Epidemiology, School of Public Health, University of Michigan, Ann Arbor, MI 48109, USA; zhaowei@umich.edu (W.Z.); kyasutak@umich.edu (K.Y.); eaugust@umich.edu (C.A.); ratliffs@umich.edu (S.M.R.); skardia@umich.edu (S.L.R.K.); needhamb@umich.edu (B.L.N.); 2 Survey Research Center, Institute for Social Research, University of Michigan, Ann Arbor, MI 48104, USA; jfaul@umich.edu (J.D.F.); dweir@umich.edu (D.R.W.); 3 Department of Human Genetics and Institute of Molecular Medicine, University of Texas Health Science Center, Houston, TX 77030, USA; Eric.Boerwinkle@uth.tmc.edu; 4 Center for Complex Disease Genomics, McKusick-Nathans Institute of Genetic Medicine, Johns Hopkins University School of Medicine, Baltimore, MD 21205, USA; aravinda@jhmi.edu; 5 Department of Epidemiology and Biostatistics, Dornsife School of Public Health, Drexel University, Philadelphia, PA 19104, USA; avd37@drexel.edu; 6 Department of Physiology and Biophysics, University of Mississippi Medical Center, Jackson, MS 39216, USA; ygao@umc.edu (Y.G.); mgriswold@umc.edu (M.E.G.); 7 Department of Epidemiology, Gillings School of Global Public Health, University of North Carolina, Chapel Hill, NC 27599, USA; gerardo_heiss@unc.edu (G.H.); kari_north@unc.edu (K.E.N.); kathryn_rose@unc.edu (K.M.R.); 8 Department of Epidemiology, Human Genetics and Environmental Sciences, School of Public Health, University of Texas Health Science Center at Houston, Houston, TX 77030, USA; alanna.c.morrison@uth.tmc.edu; 9 Department of Medicine, University of Mississippi Medical Center, Jackson, MS 39216, USA; smusani@umc.edu (S.K.M.); msims2@umc.edu (M.S.); 10 Jackson Heart Study, Jackson State University, Jackson, MS 39213, USA; smwasongwe@umc.edu; 11 Department of Epidemiology, Rollins School of Public Health, Emory University, Atlanta, GA 30322, USA; yvsun@emory.edu

**Keywords:** blood pressure, hypertension, genetics, gene-by-environment interaction, non-burden test, socioeconomic status, psychosocial factors, depression, chronic burden

## Abstract

Inter-individual variability in blood pressure (BP) is influenced by both genetic and non-genetic factors including socioeconomic and psychosocial stressors. A deeper understanding of the gene-by-socioeconomic/psychosocial factor interactions on BP may help to identify individuals that are genetically susceptible to high BP in specific social contexts. In this study, we used a genomic region-based method for longitudinal analysis, Longitudinal Gene-Environment-Wide Interaction Studies (LGEWIS), to evaluate the effects of interactions between known socioeconomic/psychosocial and genetic risk factors on systolic and diastolic BP in four large epidemiologic cohorts of European and/or African ancestry. After correction for multiple testing, two interactions were significantly associated with diastolic BP. In European ancestry participants, outward/trait anger score had a significant interaction with the *C10orf107* genomic region (*p* = 0.0019). In African ancestry participants, depressive symptom score had a significant interaction with the *HFE* genomic region (*p* = 0.0048). This study provides a foundation for using genomic region-based longitudinal analysis to identify subgroups of the population that may be at greater risk of elevated BP due to the combined influence of genetic and socioeconomic/psychosocial risk factors.

## 1. Introduction

Hypertension is a leading risk factor for cardiovascular disease and stroke and it is estimated that over 41% of adults in the U.S. will have hypertension by 2030 with a disproportionate burden among ethnic minorities [1]. Multiple lines of evidence indicate that inter-individual variation in blood pressure (BP) is influenced by both genetic [2] and non-genetic factors including socioeconomic status (SES) [3] and psychosocial factors [4]. Given that complex traits such as BP are likely shaped by multiple risk factors as well as their interactions with one another, a deeper understanding of the gene-by-socioeconomic/psychosocial factor interactions on BP may help to identify individuals that are genetically susceptible to high BP in specific social contexts.

Genome-wide association studies (GWAS) have identified significant, replicated predictors of systolic (SBP) and diastolic blood pressure (DBP) in populations of primarily European, African and Asian ancestries [5,6,7,8,9,10]. Many of the identified loci harbor genes with plausible biological roles in BP regulation [5,6,7,8,9,10]. Several physiological pathways have been proposed for these genes in the pathogenesis of hypertension, many of which are likely to be modulated by non-genetic factors. For example, the *ATP2B1* gene, which was strongly associated with both SBP and DBP [5], encodes PMCA1, a plasma membrane calcium/calmodulin-dependent ATPase that is expressed in vascular endothelium and is involved in calcium pumping from the cytosol to the extracellular compartment [11]. This signal transduction pathway is influenced by oxidative stress and inflammation [12,13], which are known to be associated with lifestyle (e.g., physical activity, diet, smoking), sociodemographic, and psychosocial factors (e.g., stress) [14,15,16,17].

In parallel, epidemiological studies have shown that socioeconomic and psychosocial factors are also related to BP levels. Several studies have linked low SES at the individual level [3], neighborhood level [18] and over the life course [19] to high BP. Possible pathways mediating these effects include influences of low SES on health behaviors such as salt intake or lack of physical activity as well as possible links between SES and stress, which has been hypothesized to be associated with BP through neuroendocrine mechanisms [20]. Psychosocial factors have also been linked to high BP, possibly through effects on behavior or direct impact on sympathetic and hypothalamic-pituitary-adrenal (HPA) axis activity [3,20,21]. For example, high levels of anger have been associated with progression from prehypertension to hypertension [20], depressive symptoms have been associated with increased SBP and DBP [22,23] as well as hypertension incidence [24] and stress has been associated with BP progression [25]. Nevertheless, findings for some psychosocial factors, such as depressive symptoms, have been inconsistent across studies and important questions remain regarding the relative importance of socioeconomic and psychosocial factors in explaining variability in BP.

The proper understanding and quantification of the etiologic roles of genes and environments in the causation of complex diseases will require consideration of gene-by-environment interactions [26,27]. Specifically, the influence of genetic predictors may be enhanced or suppressed by the presence of a certain environmental context. Likewise, the influence of environmental exposures may be modified by genetic predisposition. The investigation of gene-by-environment interactions in the genomic era remains in its infancy in part because of the lack of large population studies with rich environmental and genomic measurements as well as analytic methods that can effectively utilize the data. In addition, the majority of gene-by-environment interaction studies for complex diseases and traits such as BP have focused on demographic factors (e.g., age and body mass index (BMI)) or health behaviors (e.g., smoking, salt intake) [27] and have had cross-sectional designs. Very few have examined the socioeconomic or psychosocial environments in which genes operate and even fewer have used repeated measures data. However, the pathways through which socioeconomic and psychosocial factors are hypothesized to influence BP (which may include health behaviors but also physiologic processes such as stress response, oxidative stress, inflammation, or changes in the immune system) are likely to interact with genetic predispositions and these relationships may change over time.

In this study, we used longitudinal data from four large epidemiologic cohorts of European and/or African ancestry to investigate interactions between known socioeconomic/psychosocial and genetic risk factors on variation in BP. We used a novel genomic region-based method for repeated measures analysis, Longitudinal Gene-Environment-Wide Interaction Studies (LGEWIS) [28], to evaluate interactions rather than testing each single nucleotide polymorphism (SNP) individually. Region-based approaches such as LGEWIS may be advantageous for trans-ethnic analysis because they are able to detect interactions even in the presence of genetic heterogeneity in ancestrally diverse populations. Testing all SNPs in a region simultaneously substantially reduces multiple testing burden compared to traditional one-at-a-time SNP analysis. Furthermore, region-based analyses are able to detect the cumulative small effects of multiple SNPs within the region that may be missed by single SNP approaches that rely on the presence of high impact SNP effects. Longitudinal approaches to genetic association and gene-by-environment interaction studies that allow for varying outcome and exposure trajectories also have improved power compared to cross-sectional analysis [28,29,30]. This work represents an important first step toward comprehensively integrating social and biological factors to better understand the determinants of BP in multiple race/ethnic groups.

## 2. Materials and Methods

### 2.1. Study Cohorts

A total of 21,258 European ancestry (EA) and 8964 African ancestry (AA) participants from four population-based longitudinal cohorts of U.S. adults with up to 4 exams (time points) each were included in this analysis: the Atherosclerosis Risk in Communities Study (ARIC, 4 exams) [31], the Health and Retirement Study (HRS, 2 exams) [32], the Jackson Heart Study (JHS, 3 exams) [33,34] and the Multi-Ethnic Study of Atherosclerosis (MESA, 4 exams) [35]. A detailed description of each cohort and its characteristics are provided in the Supplementary Text and Supplementary Table S1. Participants were included in the study sample if they had genotype data and all of the following data for at least one time point: SBP and/or DBP, at least one socioeconomic/psychosocial factor, and all adjustment variables. 

This study was conducted in accordance with the Declaration of Helsinki. All participants provided informed consent prior to study participation, and data collection protocols for each cohort were reviewed by Institutional Review Boards (IRBs) at study field centers. Analysis for this project was approved by the University of Michigan IRB for Health Science and Behavioral Science (HUM00079124, HUM00012107, HUM00119419, HUM00012347).

### 2.2. Blood Pressure

For ARIC (exams 1–3), HRS and MESA, BP was measured three times and the average of the last two measures was used. For ARIC (exam 4) and JHS, two measures were taken and averaged. For participants taking antihypertensive medications, 15 and 10 mmHg were added to SBP and DBP, respectively [36]. At each exam, for each ancestry group within each cohort separately, values for SBP and DBP greater than 4 standard deviations from the mean were excluded.

### 2.3. Socioeconomic and Psychosocial Factors

Two socioeconomic and three psychosocial factors were examined: adult SES, childhood SES, outward/trait anger, depressive symptoms, and chronic burden. Lower adult SES was characterized by the respondents’ educational attainment (1 = high school degree or less). Lower childhood SES was assessed by the maximum educational attainment of either parent (1 = less than high school degree). Psychosocial factors are described below. Socioeconomic and psychosocial factors were not measured at all exams in all cohorts, so we imputed missing data to facilitate longitudinal analysis. For each cohort, the first report of adult and parental education was imputed to all time points. For psychosocial factors, missing data were imputed by carrying the most recent valid measure forward to future exams. For example, anger score was measured at exams 1 and 3 in MESA, so we imputed data at exams 2 and 4 using the values from exams 1 and 3, respectively. Supplementary Table S2 shows the exams for which each factor was measured or imputed.

#### 2.3.1. Outward/Trait Anger

Outward/trait anger scores were created from the “anger-out” and “trait anger temperament” items in the Spielberger State-Trait Anger Expression Inventory [37]. Specific questions differed somewhat across studies but included items such as, “I am quick tempered,” “I fly off the handle,” “When I get mad I say nasty things,” and “When I get mad I argue with others.” Each item was measured on a scale from 1 = “almost never” to 4 = “almost always,” and anger scores were calculated as the average item response. Items were reverse coded as necessary. Scores for respondents with missing values on more than half of the anger items were set to missing.

#### 2.3.2. Depressive Symptoms

Depressive symptom scores were created from the 20-item Center for Epidemiologic Studies—Depression (CES-D) scale [38] for MESA and JHS, the 8-item CES-D scale for HRS and the 21-item Maastricht Vital Exhaustion Questionnaire for ARIC [39]. Although the Maastricht questionnaire is not strictly a measure of depression, it has been shown to be highly correlated with measures of depression [40,41] and has been used as a measure of depression in the ARIC study [42] and for replication of GWAS results for depressive symptoms [43]. Questions from the 20-item CES-D were on a scale from 1 = “rarely or none of the time” to 4 = “most or all of the time,” corresponding to how much the respondent had experienced each symptom in the last week. Those from the 8-item CES-D were yes/no indicators of feeling the symptom much of the time in the last week (CES-D) and the Maastricht also had yes/no indicators. Items were reverse coded as necessary, such that a higher score on each scale indicates greater symptoms of depression. To create homogeneity between scores, items from the 20-item CES-D were dichotomized, collapsing values of 3 (“much of the time”) and 4 (“most or all of the time”) to correspond with the yes indicator on the other scales. Depressive symptom scores were then calculated as the average of item responses. Scores for respondents with missing values on more than half of the depression symptoms items were set to missing.

#### 2.3.3. Chronic Burden

Chronic burden scores were derived from five questions about ongoing problems for at least six (MESA and JHS) or twelve (HRS) months and included items on health, physical ailments of a spouse or child, difficulties at work, financial strain, and problems with a close relationship. Chronic burden was not measured in ARIC. Chronic burden scores were the sum of the number of ongoing problems. If more than 3 of the 5 items had missing values, scores were set to missing.

### 2.4. Genomic Regions Associated with Blood Pressure

For each study, genotyping was performed using the Affymetrix Genome-Wide Human SNP Array 6.0 (Affymetrix®, Santa Clara, CA, USA) or Illumina Infinium HumanOmni2.5 Beadchip (Illumina®, San Diego, CA, USA). Genotypes were then imputed using the 1000 Genomes Project cosmopolitan reference panel, Phase 1, version 3 (released March, 2012) [44]. Within each cohort and ancestry group separately, SNPs with minor allele frequency less than 1% or poor imputation quality (INFO or *R*^2^ < 0.8), as well as all insertion/deletions (indels), were excluded from analysis. Genetic principal components were calculated separately by ancestry group within each cohort.

A total of 33 genomic regions containing SNPs previously identified to be significant, replicated predictors of SBP and DBP in a GWAS of European ancestry [5] and a GWAS of African ancestry followed by trans-ethnic meta-analysis [6] were selected for analysis (Supplementary Table S3). If the index SNP from the GWAS was within the boundaries of a gene plus a 5kb buffer on either side, all SNPs within the gene region were selected (22 genomic regions). If the index SNP was not in a gene, all SNPs within 50 kb of the index SNP were selected (11 genomic regions). The number of SNPs selected for the genomic regions ranged from 29 to 4444 (Supplementary Table S4) and varied according to the size of the region as well as study-specific parameters such as imputation quality and allele frequencies. All position information was based on genome assembly GRCh37 and gene positions were defined by GENCODE annotation version 19 [45]. If there were multiple transcripts for a gene, the most inclusive start and stop positions were used to define the gene region. The *BAG6* genomic region was excluded from analysis in JHS, as only a single SNP in this region met criteria for analysis.

### 2.5. Adjustment Covariates

Sex and age, age^2^, and BMI at the time of BP measurement were included as adjustment variables in all models, since these were the adjustment variables in the GWAS of EA [5] and AA (except for age^2^) [6]. For each ancestry within each cohort separately, outliers greater than 4 standard deviations from the mean BMI were removed. The top 4 genetic principal components, calculated separately for each ancestry within each cohort, were included in genetic models to account for population stratification.

### 2.6. Statistical Modeling

To examine the association between each of the socioeconomic or psychosocial factors, one at a time, on SBP or DBP, we used generalized estimating equations (GEE) [46] with an exchangeable correlation structure to account for within-person correlations over time. Analyses were conducted separately for each ancestry within each cohort, using SAS v9.4 (SAS Institute, Cary, NC, USA). Associations between each of the socioeconomic/psychosocial factors and SBP or DBP were meta-analyzed using inverse variance weighting with fixed effects. Meta-analysis was conducted separately by ancestry (EA, AA) and then combining results from all ancestries (EA + AA).

To investigate the genomic region-by-socioeconomic/psychosocial factor interactions on BP, we used LGEWIS [28] in R v3.4.1 [47]. LGEWIS is a GEE-based dispersion test specifically designed for longitudinal studies to test the joint effects of SNPs/variants, or SNP/variant-by-environment interactions, within a genomic region on phenotypic variation.

In the equation below, *Y_ij_* represents the outcome variable (SBP or DBP) for *i*th subject at time *j*. *E_ij_* represents the environmental exposure (socioeconomic or psychosocial factor), and *X_ij_* represents the vector of adjustment covariates. G*_i_* = (*G_i1_, G_i2_, …, G_ip_*) represents the genotypes for the *p* SNPs within the genomic region for individual *i* and remains constant across all time points, *j*. We were primarily interested in the statistical interaction between *E_ij_* and *G_i_* on the outcome *Y_ij_*, adjusting for *X_ij_* and the main effect of *E_ij_* and *G_i_.* The LGEWIS statistical model is:

*Y_ij_* = **α′**X*_ij_* + *f*(E*_ij_*) + ***β′***G*_i_* + ***γ′***(E_ij_*G*_i_*) + *ε_ij_*,

where $\alpha^{\prime }$ = [α*_1_, ...,* α*_m_*]′ is the vector of regression coefficients for the *m* covariates and *f* (E*_ij_*) is a spline smoothing function to capture the main environmental effect, which allows for a non-linear relationship between the environment and the outcome and is thus robust to the misspecification of the main effect. ***β′*** = [*β_1_, …, β_p_*]′ is the vector of regression coefficients for the *p* observed SNPs, and ***γ′*** = [*γ_1_, …, γ_p_*]′ is the vector of regression coefficients of the interaction terms for the environment and the *p* observed SNPs. Weighted principal component analysis is used to fit the main genetic effects to avoid type I error due to overfitting. The null hypothesis of no SNP-by-environment interactions, H_0_: *γ_1_ = γ_2_=…= γ_p_ =* 0, is evaluated using an aggregated score statistic to test the overall dispersion of ***γ*** from 0. Similarly, a model without the environmental exposure or interaction terms is used to evaluate the marginal genetic effects with the null hypothesis H_0_: ***β*** = 0.

Associations between each of the genomic regions and SBP or DBP were meta-analyzed by combining *p*-values using Fisher’s method [48]. In parallel with the socioeconomic/psychosocial factor analysis, meta-analysis of the genomic region associations was conducted separately by ancestry (EA, AA) and then combining results from all ancestries (EA + AA). Previous research in gene-gene and gene-environment interaction studies shows that filtering genetic factors with at least marginal associations with the outcome of interest prior to testing for interactions can reduce multiple testing burden and increase power [49,50]. Thus, we limited assessment of gene-by-environment interactions to genomic regions that had a *p*-value < 0.2 in their marginal association with BP.

For SBP and DBP, interactions between genomic regions with marginal association *p* < 0.2 and socioeconomic/psychosocial factors were evaluated using LGEWIS. Interaction effects were meta-analyzed by combining *p*-values using Fisher’s method [48]. For each BP measure in each ancestry group (EA, AA and EA+AA), we applied a False Discovery Rate (FDR) *q*-value threshold of 0.05 to declare significance [51].

Significant genomic region-by-socioeconomic/psychosocial factor interactions were further evaluated to identify the SNPs that were most strongly contributing to the significant interaction. We evaluated the interaction between each SNP in the region and the socioeconomic/psychosocial factor of interest using GEE with an exchangeable correlation structure. Interaction effects were meta-analyzed using inverse variance weighting with fixed effects with the METAL package in R [52].

## 3. Results

### 3.1. Descriptive Statistics

Descriptive statistics at the time of first BP measurement (baseline exam) are provided for each cohort, separately by ancestry, in Table 1. Mean age ranged from 50.4 to 67.3 years and there were slightly fewer males than females (ranging from 37.4% to 47.9% male). Mean SBPs and DBPs ranged from 122.0 to 144.1 mmHg and from 73.5 to 88.8 mmHg, respectively. Cohorts with older mean ages, such as HRS, tended to have higher BPs, and BPs were also generally higher for AA than EA participants within each cohort. Lower adult and childhood SES was more prevalent in HRS and ARIC, and lower SES was also generally more prevalent in AA than EA participants within each cohort. Mean outward/trait anger scores did not differ substantially across cohorts or ancestries. Depressive symptom scores were highest in ARIC AA and lowest in MESA EA and AA. Chronic burden scores were highest in HRS and lowest in JHS.

### 3.2. Association between Socioeconomic/Psychosocial Factors and BP

Results from meta-analysis of the associations between each of the socioeconomic/psychosocial factors and BP, using data from all time points, are presented in Table 2. Lower adult SES (high school degree or less) was associated with higher SBP (2.83 and 2.77 mmHg in EA and AA, respectively) and higher DBP (0.52 and 1.10 mmHg, respectively) (all *p* < 4 × 10^−4^). Lower childhood SES was associated with higher SBP (1.83 mmHg, *p* < 5 × 10^−6^) in EA but was not associated with higher BP in AA or DBP in EA. Outward/trait anger score was associated with higher DBP in AA (*p* = 0.019). Chronic burden scores were associated with higher SBP and DBP in AA (*p* = 0.002 and 0.02, respectively) but were not associated with either SBP or DBP in EA. Depressive symptom score was also associated with higher DBP in AA (*p* = 0.002).

### 3.3. Association between Genomic Regions and BP

The association between each of the 33 genomic regions and BP was also evaluated in each ancestry group (Table 3). A total of 21, 11 and 21 genomic regions had *p* < 0.2 with SBP, and a total of 21, 10 and 23 genomic regions had *p* < 0.2 with DBP in EA, AA and the combined sample (EA + AA), respectively. These genomic regions with *p* < 0.2 were retained for subsequent genomic region-by-socioeconomic/psychosocial factor interactions. Associations tended to be less significant in AA than EA, with only 5 genomic regions for SBP and 3 genomic regions for DBP reaching a nominal significance threshold (*p* < 0.05) in the AA meta-analysis, compared to 15 and 16 regions, respectively, for EA. After correction for multiple testing, 12 and 10 regions were significantly associated (FDR *q* < 0.05) with SBP and DBP in EA, respectively, and none were significantly associated with SBP or DBP in AA.

### 3.4. Interaction between Socioeconomic/Psychosocial Factors and Genomic Regions on BP

Using the LGEWIS method, we next evaluated evidence for genomic region-by- socioeconomic/psychosocial factor interactions using genomic regions with *p* < 0.2 in the meta-analyses. After correction for multiple testing, two interactions were significantly associated with DBP (FDR *q* < 0.05, Table 4). In EA, an interaction was observed between outward/trait anger score and *C10orf107* (meta-analysis *p* = 0.0019, FDR *q* = 0.049) on DBP. The number of SNPs in the genomic region ranged from 365–400 across the three cohorts examined. *p*-values for each cohort show that the interaction was most significant in MESA (*p* = 0.0004) and HRS (*p* = 0.085). In AA, a second interaction was observed between depressive symptom score and *HFE* (meta-analysis *p* = 0.0048, FDR *q* = 0.048) on DBP. The number of SNPs in the genomic region ranged from 48 to 84 across the four cohorts examined. The interaction was most significant in ARIC (*p* = 0.0058) and MESA (*p* = 0.031). No interactions between socioeconomic/psychosocial factors and genomic regions were significantly associated with SBP in any ancestry group.

**Figure 1:**
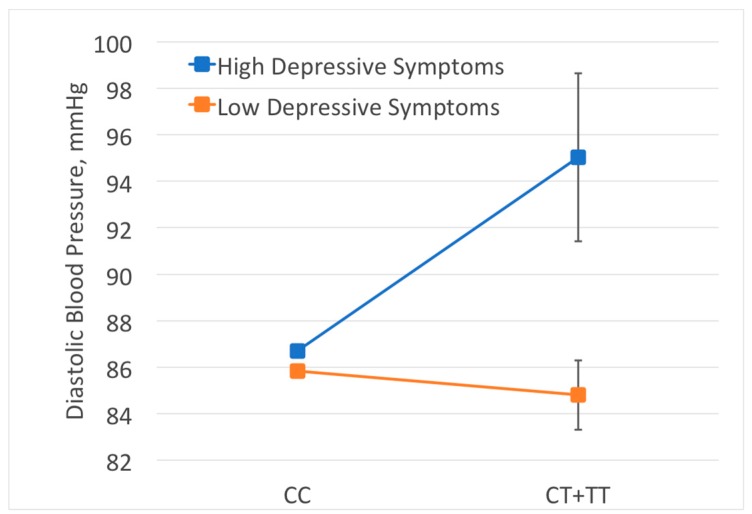
Interaction between rs147426902 and depressive symptom score on diastolic blood pressure (DBP) in African ancestry cohorts. Average predicted DBP across HRS and JHS African ancestry cohorts for those with the CC genotype at rs147426902 vs. the CT or TT genotype are presented, with depressive symptom scores at the cohort-specific 10th percentile (low depressive symptoms) or the 90th percentile (high depressive symptoms). Black bars indicate 95% confidence intervals for the average predicted DBP estimates. Due to larger sample size in the CC group, confidence intervals for this group are too small to be shown.

To identify and illustrate the SNP-by-socioeconomic/psychosocial factor interactions that may be driving the two significant genomic region-level associations that we observed, we performed meta-analysis of the interactions for each SNP within the genomic region and the socioeconomic/psychosocial factor of interest on DBP. In the EA meta-analysis of SNP-by-anger interactions for the *C10orf107* region, no single SNPs were measured in at least two cohorts and had at least a nominally significant *p*-value for the interaction with anger (*p* < 0.05). In the AA meta-analysis of SNP-by-anger interactions for the *HFE* region, the most significant SNP was rs147426902 (*p* = 0.0003), which was only present in two cohorts (HRS and JHS). Average predicted DBP for those with at least one copy of the minor allele (T) and high depressive symptoms (90th percentile of depressive symptom score within each cohort) was 9.20 mmHg higher than those with no copies of the minor allele and low depressive symptoms (10th percentile of depressive symptom score within each cohort) (Figure 1). Although SNPs were coded additively in the association analysis, we grouped those with one or two copies of the minor allele together for the purposes of generating the interaction plot (Figure 1) to ensure an adequate sample size in each cell.

## 4. Discussion

The influence of socioeconomic and psychosocial factors on BP and hypertension is widely recognized [4,53]. Advances in genomic technologies and multi-cohort collaborations are now providing evidence for the additional influence of hundreds of genes [5,6,7,8,9,10]. However, most of the heritability of BP remains unexplained, likely in part due to the presence of gene-by-environment interactions [27,54]. A deeper understanding of the context-dependent genetic effects on BP may yield insight into the biological mechanisms of hypertension etiology and facilitate personalized medicine approaches to BP control that may include lifestyle modifications, more aggressive prevention or treatment approaches, and/or pharmacogenomics. Further, a more complete understanding of the similarities and differences in context-dependent effects across multiple race/ethnic groups may help in the development of effective strategies for reducing health disparities in hypertension. 

To our knowledge, this study is one of the first to meta-analyze the effects of socioeconomic and psychosocial factors on BP in multiple cohorts of European and African ancestry. Consistent with prior literature [3,19,55], we found that lower adult SES, as measured by having less than a high school degree, was associated with increased BP. Lower childhood SES was most strongly associated with SBP in EA but was not associated with BP in AA. Of the three psychosocial stressors evaluated (anger, depressive symptoms, and chronic burden), all were associated with both SBP and DBP in AA participants, except for depressive symptoms with SBP. However, they were not significantly associated with BP in EA. All observed associations were in the expected direction (lower SES and childhood SES and greater anger, depressive symptoms, and chronic burden were associated with higher BPs).

A previous study in EA and AA ARIC participants found that high levels of trait anger were associated with progression from prehypertension to hypertension [20]. A recent study in AA from JHS [25] also found outwardly-expressed anger is associated with BP stage progression. Hostility, a trait closely related to anger, was associated with hypertension risk in the Coronary Artery Risk in Young Adults study (CARDIA) as well [56]. In this study, outward/trait anger associated with continuous measures of SBP in AA, with each increase of 1 unit on the outward/trait anger score (range 0–4) conferring an increase of 0.85 mmHg. For AA, a 1 unit increase in anger was also associated with a 0.68 mmHg increase in DBP. The strong relationship between anger and BP in this study sample is perhaps expected, since ARIC and JHS comprise two of the four cohorts meta-analyzed here. In EA, although the relationship between anger and BP was in the expected direction, the effect was not significant. Biological mechanisms for this relationship may include sympathetic nervous system hyperactivity and arousal due to anger and psychological stress [20].

Although some studies have demonstrated increased risk of hypertension [24,57,58], BP progression (an increase in BP stage) [25], or increases in BP [22,23] with depression/depressive symptoms, other studies show inverse [59] or no relationships [4]. Differences in findings may be due to study design (duration of follow-up, measurement of depression, and age or race/ethnic composition of the study sample). In this study, repeated measures analysis showed no association between depressive symptoms and either SBP or DBP in EA and only a weak relationship with SBP in AA. However, the relationship between depressive symptom score and DBP was significant in AA (*p* = 0.002), with a 10% increase in depressive symptom score associated with a 0.18 mmHg increase in DBP. Potential mechanisms for the relationship between depression and hypertension include both behavioral (e.g., smoking, physical activity, adherence to antihypertensive medication) and biological (e.g., inflammation, altered night-time BP dipping as a result of insomnia) mechanisms [60].

We used a novel genomic region-based, repeated measures approach to assess the relationship between 33 genomic regions known to be associated with SBP, DBP, or both. These regions were identified in a GWAS meta-analysis of EA (29 regions) [5] and a GWAS meta-analysis of AA followed by trans-ethnic meta-analysis (an additional 4 regions) [6]. Of these 33 regions, 25 (75%) had a marginal association *p*-value of < 0.2 for SBP and/or DBP in our meta-analysis of EAs, with the majority of these (17 regions) achieving *p <* 0.2 in both. In AA, a smaller number of regions (16, 48%) had a marginal association *p*-value < 0.2, including only 5 regions with *p <* 0.2 for both SBP and DBP. These regions with marginal *p <* 0.2 were carried forward for genomic region-by-psychosocial factor interaction analysis. However, only 12 and 10 regions in EA were significant with SBP and DBP, respectively, at a False Discovery Rate of 5%. No regions were significant after multiple testing correction in AA, likely because most of the regions were identified in meta-analysis of EA only, as well as the significantly smaller sample size of AA compared to EA participants in this study.

Gene-by-environment interaction studies on BP and related traits are now being reported and catalogued [27,61], including large-scale genome-wide analyses of genetic interactions with lifestyle factors (alcohol consumption [62], smoking [63,64,65], sodium intake [66], age [67], and BMI [68]). To our knowledge, only one genome-wide study has examined interactions between socioeconomic or psychosocial factors and BP. In this study of 3836 participants from the Framingham Heart Study (FHS), two genome-wide significant and three suggestive gene-by-education interactions on SBP (1 interaction), DBP (3 interactions) and pulse pressure (1 interaction) were identified [69]. Although the 5 genes identified were all biologically related to BP, none were identified in GWAS studies used to select genomic regions for evaluation in this study, so they were not included in our analysis. We also identified one study that examined interactions between polymorphisms in biological candidate genes and socioeconomic/psychosocial factors on BP. In this study of 208 AAs, neighborhood SES was found to interact with glucocorticoid receptor polymorphisms to influence cortisol levels but not BP [70]. Finally, in the paper describing the LGEWIS method, He et al. investigated interactions between genes known to influence BP and perceived or geographic information system (GIS)-based measures of healthy food and physical activity environment in MESA participants [28]. Interactions were observed for SBP between the genomic region indexed by rs10850411 and perceived healthy food availability in EA, and between the *CACNB2* genomic region and density of recreational facilities in Hispanic Americans. Although we evaluated both of these genomic regions in our study, we did not observe interactions between these regions and socioeconomic/psychosocial factors on BP.

In this multi-cohort study examining socioeconomic/psychosocial factor interactions with genomic regions identified through large, replicated BP GWAS, we observed two significant interactions after correction for multiple testing. Outward/trait anger was found to have an interaction with the *C10orf107* gene region on DBP in EA. *C10orf107*, or *CABCOCO1*, is a relatively uncharacterized gene but the mouse homolog encodes a protein with a predicted coiled-coil domain and a CLAMP motif with a leucine zipper domain that has calcium-binding activity [71]. Results of tissue-specific gene expression patterns from the GTEx Analysis Release V6p (dbGaP Accession phs000424.v6.p1) [72] showed that this protein is most highly expressed in the testis, spinal cord and brain tissues, pituitary gland, prostate and lung. Further research is necessary to characterize the biological mechanisms that underlie the interaction between *C10orf107* and anger on DBP. To ensure that the interaction was not detected due to gene-environment correlation, we used LGEWIS to assess the association between the *C10orf107* genomic region and anger (*p* > 0.1 in all cohorts, indicating no gene-environment correlation). Single SNP analysis in this region revealed no SNPs that appeared to be driving this association in multiple cohorts. This finding in EA illustrates the ability of genomic region-based analysis techniques to detect interaction signals that reflect a shift in the entire distribution of interaction effects across the genomic region, due to the presence of multiple SNPs that have small influences, even when no SNPs with high-impact interaction effects are present.

We also identified an interaction between the *HFE* gene region and depression symptom score on DBP in AA. *HFE* encodes a membrane protein that regulates iron binding, which influences absorption of iron in the small intestine and recycling of iron in macrophages [73] and has also been shown to demonstrate immune-related activities that bridge adaptive and innate immunity [74]. Mutations in *HFE* can cause hereditary hemochromatosis (HH) [75], characterized by iron accumulation that can lead to tissue damage. Hemochromatosis may be associated with BP through increased serum ferritin (iron) levels that alter heart morphology or cause metabolic abnormalities such as insulin resistance [76,77]. Early signs of hemochromatosis include fatigue, malaise, joint pain and swelling and enlarged liver [78]. In a recent study of 395 subjects with *HFE*-related HH, 41% of the HH patients meeting the criteria for fibromyalgia syndrome, characterized by chronic joint pain and fatigue, also met the criteria for depression. To ensure that the interaction observed in this study was not due to gene-by-environment correlation, we tested the *HFE* genomic region for association with depressive symptom score in each of the AA cohorts using LGEWIS. None of the cohorts demonstrated significant association (all *p* > 0.1, data not shown), so we concluded that the interaction was not due to correlation between *HFE* and depressive symptoms.

This study is not without limitations. The LGEWIS method only assesses the significance of the interaction term between the genomic region and the socioeconomic/psychosocial factor. However, in some circumstances including gene discovery, it may be optimal to test the joint effects of the genomic region and the interaction simultaneously. This approach has been favored by large consortia now investigating gene-by-environment interactions on a genome-wide scale [79]. Further work is needed to expand the repeated-measures genomic region-based methods to incorporate the analysis of joint effects. Another limitation is that this study included only 33 of the genomic regions that have been identified as being associated with BP. However, recent GWAS have increased the number of loci to over 100 [7,8,9,10] and exploring interactions with these newly discovered loci is an important direction for future research. In addition, the 33 genomic regions were selected through GWAS meta-analyses that contained the cohorts analyzed in this study. The ARIC EA participants were included in the discovery sample (*N* = 69,395) of the GWAS meta-analysis in EA only (total *N* > 200,000 EA) [5], and all four of the AA cohorts were included in the discovery sample (*N* = 29,378) of the trans-ethnic meta-analysis (total *N* > 120,000) [6]. From a genetic perspective, another limitation is that this study only evaluated common SNPs; however, this line of research could be expanded to examine interactions in these gene regions using measures of rare, potentially functional variants such as exome or whole genome sequence data.

From a phenotypic perspective, although we were able to successfully harmonize socioeconomic/psychosocial factors in this study, there is heterogeneity across the cohorts in the measurement of these factors. This is an ongoing challenge for gene-environment interaction studies [80], emphasizing the need standardized phenotype measures such as those catalogued in the Phoenix Toolkit (www.phenxtoolkit.org). In addition, we used educational attainment as our sole marker of SES, which is only one facet of individual-level SES and may be confounded with other factors that influence health (such as living in a rural vs. urban area). Educational attainment and the psychosocial factors evaluated in this study may also be confounded by other unmeasured exposures such as smoking, alcohol consumption and physical activity; however, these lifestyle factors may also act as mechanisms by which socioeconomic/psychosocial factors influence BP. Although all cohorts retained over 70% of participants over the course of the study, attrition was more extreme for AA (vs. EA), lower SES (vs. higher SES), and older participants. To help alleviate concerns about bias due to attrition, analyses were controlled for age and sex and were stratified by ethnicity. Sensitivity analyses indicated that further adjustment for adult SES did not attenuate the significant interaction findings (data not shown). We chose to account for antihypertensive use by adding a constant value (+15/10 mmHg) to BP; however, we conducted a sensitivity analysis to evaluate whether an alternative method (using original BP measures and including antihypertensive use as a covariate in all models) influenced they key findings. Effect sizes for significant associations between psychosocial factors and BP, as well *p*-values for the two significant genomic region-by-psychosocial factor interactions on DBP, did not change appreciably using this alternative method (data not shown). Finally, we included BMI as an adjustment variable in all models. This approach, used by the discovery GWASs [5,6], reduces residual BP variance and boosts power to detect interaction effects; however, it reduces power to detect interactions mediated at least in part by changes in BMI.

We detected two interactions in this study but their effect on BP is small, and although these findings were statistically significant, it is still possible that they were due to chance. Therefore, the clinical relevance of these findings has yet to be determined. Further, although our multiple testing correction accounted for the number of genes we evaluated for interaction, we did not account for testing multiple socioeconomic/psychosocial factors within each ancestry group. Since the socioeconomic/psychosocial factors are correlated, a Bonferroni approach would have been conservative in this instance. However, if we had followed this more stringent multiple testing approach, the interactions we detected would have been attenuated. Although we included four cohorts in this study, we also had limited power to detect effects, which may account for the lack of significance of the associations between some of the genomic regions and BP, especially in AA. Lack of power may also have contributed to inconsistent findings across ancestry groups, both for genomic region associations with BP as well as interactions.

Despite these limitations, this study has several notable strengths including the use of novel genomic region-based methods that allow for repeated measures analysis. Genomic region-based methods to assess interaction are advantageous because they reduce the multiple testing burden compared to testing each SNP individually, they allow for the detection of interactions that are driven by multiple SNPs within a genomic region rather than those being driven by a single genetic factor, and they may also be more suited for trans-ethnic studies because they do not rely on SNPs to have similar linkage disequilibrium patterns across ancestry groups. Meta-analysis across multiple cohorts increases the credibility and generalizability of findings, yet additional replication will be necessary to further confirm these interactions.

## 5. Conclusions

In this meta-analysis of gene-by-socioeconomic/psychosocial factor interactions using data from four large epidemiologic cohorts of European and/or African ancestry participants, we find evidence for interactions that influence DBP. Although effect sizes for these interactions are modest and replication in independent cohorts is needed, this study provides a foundation for using genomic region-based longitudinal analysis to identify subgroups of the population that are at greater risk of elevated BP due to the combined influence of genetic and socioeconomic/psychosocial risk factors. For some groups, this approach may be clinically relevant for reducing risk of hypertension in this era of precision medicine. Integrating genomic and social approaches may also lend insight into effective strategies for reducing socioeconomic and race/ethnic health disparities in hypertension. 

## Figures and Tables

**Table 1:** Baseline ^a^ descriptive statistics for four cohort studies.

	ARIC		HRS		JHS		MESA	
	Total N ^b^	Mean (SD) or N (%)	Total N ^b^	Mean (SD) or N (%)	Total N ^b^	Mean (SD) or N (%)	Total N ^b^	Mean (SD) or N (%)
*European Ancestry*								
Gender (Male)	9274	4379 (47)	9441	4037 (43)	--	--	2518	1205 (48)
Age, years	9274	54.3 (5.7)	9441	67.3 (10.9)	--	--	2518	62.7 (10.2)
BMI, kg/m^2^	9274	26.9 (4.7)	9441	28.9 (5.6)	--	--	2518	27.7 (5.0)
SBP, mmHg ^c^	9265	122.0 (19.3)	9440	136.8 (22.5)	--	--	2516	128.4 (23.0)
DBP, mmHg ^c^	9266	74.1 (11.4)	9434	83.7 (12.3)	--	--	2518	73.5 (11.3)
Lower Adult SES ^d^	9262	4826 (52)	9420	4704 (50)	--	--	2510	549 (22)
Lower Childhood SES ^e^	7338	3877 (53%)	8462	3204 (38%)	--	--	2359	677 (29)
Outward/Trait Anger Score	8920	1.6 (0.4)	8574	1.5 (0.5)	--	--	2509	1.5 (0.3)
Depressive Symptom Score	8924	0.2 (0.2)	9441	0.2 (0.2)	--	--	2507	0.1 (0.1)
Chronic Burden Score	--	--	5167	1.7 (1.3)	--	--	2510	1.1 (1.1)
*African Ancestry*								
Gender (Male)	3155	1182 (38)	2060	770 (37)	2117	836 (40)	1608	743 (46)
Age, years	3155	53.4 (5.8)	2060	63.2 (10.5)	2117	50.4 (12.0)	1608	62.3 (10.1)
BMI, kg/m^2^	3155	29.5 (5.8)	2060	30.6 (6.5)	2117	32.1 (7.3)	1608	30.1 (5.8)
SBP, mmHg ^c^	3146	134.6 (22.4)	2060	144.1 (24.3)	2115	131.7 (19.5)	1606	139.1 (24.5)
DBP, mmHg ^c^	3153	84.1 (13.4)	2059	88.8 (13.7)	2117	80.8 (10.3)	1607	79.5 (11.7)
Lower Adult SES ^d^	3148	1931 (61)	2055	1294 (63)	2113	637 (30)	1595	497 (31)
Lower Childhood SES ^e^	2014	1541 (77%)	1759	1025 (58%)	1824	855 (47)	1412	623 (44)
Outward/Trait Anger Score	2885	1.6 (0.4)	1502	1.5 (0.5)	1380	1.6 (0.4)	1593	1.4 (0.3)
Depressive Symptom Score	2887	0.3 (0.2)	2058	0.2 (0.3)	1429	0.2 (0.2)	1590	0.1 (0.1)
Chronic Burden Score	--	--	982	2.2 (1.4)	1506	0.9 (1.2)	1593	1.2 (1.2)

ARIC: Atherosclerosis Risk in Communities; HRS: Health and Retirement Study; JHS: Jackson Heart Study; MESA: Multi-Ethnic Study of Atherosclerosis; BMI: body mass index; SBP: systolic blood pressure; DBP: diastolic blood pressure; SES: socioeconomic status. ^a^ Baseline was defined as the first exam at which each measure was taken. For ARIC, all measures were taken at Exam 1 except for anger and depressive symptoms scores, which were first measured at Exam 2. For HRS, blood pressure (BP) was measured at alternating exam dates (during the face-to-face exam only), so values for all measures reflect the corresponding year at which BP was first measured (2006, 2008, or 2010). Chronic burden was not measured in HRS in 2008. For JHS, all measures were taken at Exam 1 except chronic burden, which was first measured at Exam 3. For MESA, all measures were taken at Exam 1. ^b^ Total N reflects the number of non-missing values with valid measures on SBP or DBP, gender, age and BMI. ^c^ For participants taking antihypertensive medications, 15 and 10 mmHg were added to SBP and DBP, respectively. ^d^ High school degree or less. ^e^ Parental education less than high school degree.

**Table 2:** Meta-analysis of the association between each socioeconomic or psychosocial factor and blood pressure.

	SBP		DBP	
	Beta	*p*-Value	Beta	*p*-Value
*European Ancestry*				
Lower Adult SES ^a^	2.83	<5 × 10^−6^	0.52	3.6 × 10^−4^
Lower Childhood SES ^b^	1.83	<5 × 10^−6^	0.12	0.431
Outward/Trait Anger Score	0.42	0.097	0.13	0.375
Depressive Symptom Score	0.32	0.566	−0.24	0.459
Chronic Burden Score	−0.02	0.871	−0.03	0.629
*African Ancestry*				
Lower Adult SES ^a^	2.77	<5 × 10^−6^	1.10	<5 × 10^−6^
Lower Childhood SES ^b^	0.52	0.268	−0.04	0.887
Outward/Trait Anger Score	0.95	0.027	0.57	0.019
Depressive Symptom Score	1.36	0.186	1.80	0.002
Chronic Burden Score	0.64	0.002	0.25	0.020
*European Ancestry + African Ancestry*				
Lower Adult SES ^a^	2.81	<5 × 10^−6^	0.68	<5 × 10^−6^
Lower Childhood SES ^b^	1.49	<5 × 10^−6^	0.08	0.545
Outward/Trait Anger Score	0.56	0.011	0.24	0.050
Depressive Symptom Score	0.56	0.255	0.25	0.377
Chronic Burden Score	0.16	0.142	0.05	0.408

^a^ High school degree or less. ^b^ Parental education less than high school degree. Generalized estimating equations were used to model the effect of each socioeconomic/psychosocial factor separately on SBP or DBP, adjusting for sex and age, age^2^, and BMI at the time of BP measurement. Betas and *p*-values represent beta coefficients (effect estimates) for the socioeconomic/psychosocial factors from the inverse variance-weighted fixed effects meta-analysis across cohorts and their corresponding *p*-values.

**Table 3:** *p*-values from meta-analysis of the association between each genomic region and blood pressure.

	SBP			DBP		
Genomic Region	EA	AA	EA + AA	EA	AA	EA + AA
	*p*-Value	*p*-Value	*p*-Value	*p*-Value	*p*-Value	*p*-Value
*ARHGAP42*	**0.1157**	0.9122	0.4840	0.7648	0.6353	0.8013
*ATP2B1*	<5 × 10^−6^	0.9550	<5 × 10^−6^ *	0.0001 *	0.6745	0.0015 *
*BAG6*	0.5830	0.6999	0.7427	0.6535	0.5613	0.6999
*C10orf107*	0.2420	0.7852	0.5516	0.0094 *	**0.0462**	0.0032 *
*CACNB2*	0.0032 *	0.6633	**0.0297**	**0.1055**	0.4553	**0.1948**
*CSK*	0.0002 *	**0.1863**	0.0006 *	0.0015 *	0.5212	0.0115 *
*FES*	0.0057 *	0.9461	**0.1008**	0.0049 *	0.5335	**0.0289**
*GOSR2*	0.0005 *	0.2659	0.0019 *	**0.0413**	**0.1341**	**0.0298**
*GUCY1A3*	0.2639	**0.0055**	0.0093 *	0.6453	**0.0645**	**0.1662**
*HFE*	**0.0549**	**0.0242**	0.0077 *	**0.0355**	**0.1282**	**0.0254**
*MECOM*	0.0043 *	0.3570	**0.0155**	0.0001 *	**0.1986**	0.0003 *
*MTHFR*	**0.0236**	0.8904	**0.1973**	0.0003 *	0.9941	**0.0191**
*NT5C2*	0.0111 *	**0.0478**	0.0038*	0.0025*	0.6324	**0.0228**
*PLCE1*	0.6174	**0.0817**	**0.1873**	0.3960	0.3966	0.4036
*PLEKHA7*	**0.1594**	**0.1885**	**0.1151**	0.2518	0.2767	0.2230
*PLEKHG1*	**0.0256**	0.6792	**0.1270**	0.0044 *	0.3302	**0.0142**
rs10850411	0.3666	0.2214	0.2460	**0.0688**	0.5504	**0.1816**
rs1173771	0.2511	0.5098	0.3729	0.7050	0.6744	0.7945
rs11953630	0.3041	0.4429	0.3722	**0.0352**	0.4752	**0.0984**
rs13082711	0.0082 *	0.7951	**0.0798**	**0.0654**	0.2571	**0.0794**
rs13209747	0.5173	**0.1037**	**0.1869**	**0.0171**	0.5513	**0.0725**
rs1327235	**0.0445**	**0.0356**	**0.0092**	0.0037 *	**0.0613**	0.0019 *
rs1458038	0.0083 *	0.3549	**0.0249**	**0.0235**	0.4374	**0.0677**
rs17428471	0.0007 *	**0.0022**	1.7 × 10^−5^ *	**0.0435**	**0.0690**	**0.0166**
rs2932538	**0.0704**	0.8772	0.3506	0.4944	0.9010	0.8393
rs4373814	0.0160 *	0.4816	**0.0582**	0.2120	0.5541	0.3639
rs7129220	0.4061	0.8965	0.7845	0.4627	0.6577	0.6413
*SH2B3*	**0.0581**	**0.1463**	**0.0423**	0.0048 *	0.2540	0.0111 *
*SLC39A8*	0.2630	0.4592	0.3505	0.4093	0.3212	0.3527
*SOX6*	0.0024 *	0.7271	**0.0289**	**0.0574**	**0.0306**	0.0099 *
*ULK4*	0.3536	0.6047	0.5235	0.2123	**0.0626**	**0.0571**
*ZNF652*	0.7850	**0.0840**	0.2508	0.2715	**0.0047**	0.0085 *
*ZNF831*	**0.1331**	0.8755	0.4803	**0.1674**	0.4666	0.2681
Number of genes with *p* < 0.2	21	11	21	21	10	23
Number of genes with FDR *q* < 0.05	12	0	7	10	0	8

EA = European ancestry; AA = African ancestry. Longitudinal Gene-Environment-Wide Interaction Studies (LGEWIS) was used to model the effect of each gene region separately on SBP or DBP, adjusting for sex, top 4 genetic principal components and age, age^2^, and BMI at the time of BP measurement. *p*-values are Fisher’s combined *p*-value across cohorts for the meta-analysis of the association between genomic region and BP. *p*-values < 0.2 are in bold, indicating associations that were further explored for interactions between the genomic region and each socioeconomic/psychosocial factor. * Indicates that genomic region is significantly associated with FDR *q* < 0.05.

**Table 4:** *p*-values for significant interactions between psychosocial factors and genomic regions on diastolic blood pressure.

Ancestry	Psychosocial Factor	Genomic Region	Number of SNPs ^a^	ARIC *p*-Value	HRS *p*-Value	JHS *p*-Value	MESA *p*-Value	Meta-Analysis *p*-Value (FDR *q*) ^b^
EA	Outward/Trait Anger Score	*C10orf107*	365–400	0.801	0.085	N/A	0.0004	0.0019 (0.049)
AA	Depressive Symptom Score	*HFE*	46–84	0.006	0.162	0.550	0.031	0.0048 (0.048)

EA = European ancestry; AA = African ancestry. LGEWIS was used to model interactions between socioeconomic/psychosocial factors and genomic regions on SBP or DBP, adjusting for sex, top 4 genetic principal components and age, age^2^, and body mass index (BMI) at the time of BP measurement. ^a^ Range of the number of SNPs included in the genomic region across cohorts. ^b^ Fisher’s combined *p*-value across cohorts for the meta-analysis of the association between the interaction and DBP and corresponding FDR q-value.

## Supplementary Materials

The following are available online at www.mdpi.com/1660-4601/14/12/1596/s1, Supplementary Text: Study cohort and blood pressure measurement descriptions, Table S1: Study cohort characteristics, Table S2: Demographic, blood pressure, socioeconomic and psychosocial measures for each cohort, Table S3: Genomic regions selected for analysis, Table S4: Number of SNPs in genomic regions selected for analysis.
