# Supplementary material for: Gene-by-Psychosocial Factor Interactions Influence Diastolic Blood Pressure in European and African Ancestry Populations: Meta-Analysis of Four Cohort Studies
Source: Int J Environ Res Public Health. 2017 Dec 18;14(12):1596. doi: 10.3390/ijerph14121596 (PMC5751013; doi:10.3390/ijerph14121596)
Supplement: Supplementary file 1 [file ijerph-14-01596-s001.zip › Smith IJERPH 10_01_2017 SuppTable4.pdf]

Supplemental Table 4: Number of SNPs in genomic regions selected for analysis

Genomic Region	European Ancestry			African Ancestry				Average
	ARIC	HRS	MESA	ARIC	HRS	MESA	JHS	
ARHGAP42	1269	1341	1230	1904	1874	1852	1246	1531
ATP2B1	185	216	186	350	382	338	183	263
BAG6 [a]	45	46	39	35	40	44	1	36
C10orf107	370	400	365	498	499	476	264	410
CACNB2	1504	1578	1432	2510	2552	2158	1438	1882
CSK	33	42	30	72	85	53	33	50
FES	45	52	43	92	108	45	29	59
GOSR2	364	385	337	478	502	448	238	393
GUCY1A3	194	219	184	336	405	263	168	253
HFE	57	59	54	84	83	78	46	66
MECOM	1530	1643	1429	2217	2698	2158	1378	1865
MTHFR	134	140	134	183	204	163	126	155
NT5C2	256	272	253	326	365	317	166	279
PLCE1	606	656	594	1230	1353	1179	723	906
PLEKHA7	743	794	720	1208	1298	1101	711	939
PLEKHG1	846	939	802	1406	1553	1166	840	1079
rs10850411	428	446	411	673	737	574	345	516
rs1173771	244	258	240	503	571	473	321	373
rs11953630	317	318	314	489	556	477	321	399
rs13082711	294	308	286	451	464	422	245	353
rs13209747	174	200	164	396	391	360	229	273
rs1327235	356	378	344	499	611	414	316	417
rs1458038	301	317	281	523	610	389	276	385
rs17428471	308	339	284	431	507	361	221	350
rs2932538	158	211	149	197	374	219	133	206
rs4373814	374	416	363	608	628	514	352	465
rs7129220	300	331	284	434	478	384	229	349
SH2B3	37	41	32	109	139	118	66	77
SLC39A8	518	556	509	941	966	898	555	706
SOX6	1209	1324	1278	2714	2823	2469	1375	1885
ULK4	2072	2301	1972	4289	4444	4088	2629	3114
ZNF652	184	190	171	322	323	297	193	240
ZNF831	233	242	196	382	450	289	214	287

[a] JHS was excluded from analysis of the *BAG6* genomic region, due to the low number of SNPs meeting analysis criteria.
