# Supplementary material for: Gene-by-Psychosocial Factor Interactions Influence Diastolic Blood Pressure in European and African Ancestry Populations: Meta-Analysis of Four Cohort Studies
Source: Int J Environ Res Public Health. 2017 Dec 18;14(12):1596. doi: 10.3390/ijerph14121596 (PMC5751013; doi:10.3390/ijerph14121596)
Supplement: Supplementary file 1 [file ijerph-14-01596-s001.zip › Smith IJERPH 10_01_2017 SuppText.pdf]

Study cohort and blood pressure measurement descriptions

The Atherosclerosis Risk in Communities (ARIC) Study [1] is an ongoing NHLBI population-based cohort. Briefly, participants aged 45 to 64 years at baseline were recruited from four communities: Forsyth County, North Carolina; Jackson, Mississippi; Minneapolis, Minnesota; and Washington County, Maryland. A total of 15,792 individuals, predominantly of European and African ancestry, participated in the baseline examination in 1987-1989, with three additional triennial follow-up examinations and a fifth exam in 2011-2013. This study considers blood pressure measurements from ARIC visits (exams) 1-4. Blood pressure was measured using a random zero sphygmomanometer. Three measurements were taken at Exams 1 through 3, and two at Exam 4. For each exam, the average of the last two measurements was used for analysis.

The Health and Retirement Study (HRS) [2] is a longitudinal panel study that surveys a representative sample of more than 26,000 Americans over the age of 50 every two years with over-sampling in minority populations, using alternating face-to-face and telephone interviews. This study explores the changes in labor force participation and the health transitions that individuals undergo toward the end of their work lives and in the years that follow. Since its launch in 1992, the study has collected information about income, education, disability, physical health and functioning, and cognitive functioning. The sample for this analysis consisted of people who provided saliva samples for genotyping in 2006, 2008, and 2010. Blood pressure was assessed during the face-to-face interviews starting in 2006 using an Omron automated blood pressure device. At each exam, three measurements were taken and the average of the last two measurements was used for analysis.

The Jackson Heart Study (JHS) [3,4] is a prospective population-based study to seek the causes of the high prevalence of common complex diseases among African Americans in the Jackson, Mississippi metropolitan area, including cardiovascular disease, type-2 diabetes, obesity, chronic kidney disease, and stroke. During the baseline examination period (2000-2004) 5,306 self-identified African Americans were recruited from four sources, including (1) randomly sampled households from a commercial listing; (2) ARIC participants; (3) a structured volunteer sample that was designed to mirror the eligible population; and (4) a nested family cohort. Unrelated participants were between 35 and 84 years old, and members of the family cohort were ≥ 21 years old when consent for genetic testing was obtained and blood was drawn for DNA extraction. Based on DNA availability, appropriate informed consent, and genotyping results that met quality control procedures, genotype data were available for 3,027 individuals, including 885 who are also ARIC participants. In the current study, JHS participants who were also enrolled in the ARIC study were analyzed with the ARIC dataset —for this reason, the JHS data set analyzed here is defined as 2,145 individuals, of whom 2,129 are included in the current analysis. Blood pressure was measured using a random zero sphygmomanometer. At each exam, two measurements were taken and the average was used for analysis.

The Multi-Ethnic Study on Atherosclerosis (MESA) [5] is a longitudinal study of the characteristics of subclinical cardiovascular disease and the risk factors that predict progression to clinically overt cardiovascular disease. MESA began in 2002 with a sample of 6,814 asymptomatic men and women aged 45-84 recruited in six communities: Baltimore City and Baltimore County, MD; Chicago, IL; Forsyth County, NC; Los Angeles County, CA; New York, NY; and St. Paul, MN. Approximately 38% of the participants are non-Hispanic white, 28% African American, 22% Hispanic, and 12% Asian. Five follow-up examinations and multiple ancillary studies have been conducted since MESA's inception, and a sixth examination is currently underway. Data collected includes traditional coronary disease risk factors, socio-demographic factors, lifestyle factors, and psychosocial factors. Blood

pressure was measured using a Dinamap automated blood pressure device. At each exam, three measurements were taken and the average of the last two measurements was used for analysis.

References

1. The ARIC Investigators. The Atherosclerosis Risk in Communities (ARIC) Study: Design and Objectives. *Am. J. Epidemiol.* **1989**, *129*, 687-702.
2. Sonnega, A.; Faul, J.D.; Ofstedal, M.B.; Langa, K.M.; Phillips, J.W.; Weir, D.R. Cohort Profile: The Health and Retirement Study (HRS). *Int. J. Epidemiol.* **2014**, *43*, 576-585.
3. Taylor, H.A.,Jr; Wilson, J.G.; Jones, D.W.; Sarpong, D.F.; Srinivasan, A.; Garrison, R.J.; Nelson, C.; Wyatt, S.B. Toward Resolution of Cardiovascular Health Disparities in African Americans: Design and Methods of the Jackson Heart Study. *Ethn. Dis.* **2005**, *15*, S6-4-17.
4. Wilson, J.G.; Rotimi, C.N.; Ekunwe, L.; Royal, C.D.; Crump, M.E.; Wyatt, S.B.; Steffes, M.W.; Adeyemo, A.; Zhou, J.; Taylor, H.A.,Jr *et al.* Study Design for Genetic Analysis in the Jackson Heart Study. *Ethn. Dis.* **2005**, *15*, S6-30-37.
5. Bild, D.E.; Bluemke, D.A.; Burke, G.L.; Detrano, R.; Diez Roux, A.V.; Folsom, A.R.; Greenland, P.; Jacob, D.R.,Jr; Kronmal, R.; Liu, K. *et al.* Multi-Ethnic Study of Atherosclerosis: Objectives and Design. *Am. J. Epidemiol.* **2002**, *156*, 871-881.
