# Supplementary material for: Gene-by-Psychosocial Factor Interactions Influence Diastolic Blood Pressure in European and African Ancestry Populations: Meta-Analysis of Four Cohort Studies
Source: Int J Environ Res Public Health. 2017 Dec 18;14(12):1596. doi: 10.3390/ijerph14121596 (PMC5751013; doi:10.3390/ijerph14121596)
Supplement: Supplementary file 1 [file ijerph-14-01596-s001.zip › Smith IJERPH 10_01_2017 SuppTable1.pdf]

Supplemental Table 1: Study cohort characteristics

Cohort (Acronym)	PMID	Sample Size[a]	Number of Exams[b]	Exam Dates	Genotyping Platform	Genotyping Exclusions Prior to Imputation	Imputation Software	Imputation Panel
Atherosclerosis Risk in Communities Study (ARIC)	2646917	9,292 EA; 3,169 AA	4	1987-1996	Affymetrix Genome-Wide Human SNP Array 6.0	SNPs with MAF<0.01, call rate<95%, HWE $p<10^{-5}$; individuals with gender mismatch, genetic outliers, first-degree relatives, concordance check with Taqman data>20%	IMPUTE v2	1000 Genomes Project cosmopolitan reference panel, phase 1, version 3
Health and Retirement Study (HRS)	24671021	9,441 EA; 2,060 AA	2	2006-2012	Illumina Infinium HumanOmni 2.5 BeadChip	SNPs with call rate <98%, HWE $p<10^{-4}$, chromosomal anomalies; individuals with call rate <98%, unresolved gender mismatches, first degree relatives, genetic outliers	IMPUTE v2	1000 Genomes Project cosmopolitan reference panel, phase 1, version 3
Jackson Heart Study (JHS)	16320381, 16317983	2,126 AA	3	2000-2013	Affymetrix Genome-Wide Human SNP Array 6.0	SNPs with MAF<0.01 and genotyping success rate <10%, HWE $p<10^{-6}$, Mendelian errors, unresolved gender mismatches, genetic outliers	MaCH (version 1.0.16)	1000 Genomes Project cosmopolitan reference panel, phase 1, version 3
Multi-Ethnic Study of Atherosclerosis (MESA)	12397006	2,525 EA; 1,609 AA	4	2000-2007	Affymetrix Genome-Wide Human SNP Array 6.0	SNPs with call rate <95%, heterozygosity>53%; individuals with call rate<95%, unresolved gender mismatches, cryptic duplicates, genetic outliers	IMPUTE v2	1000 Genomes Project cosmopolitan reference panel, phase 1, version 3

[a] Sample size reflects number of participants with genotype data and all of the following data for at least one time point: SBP and/or DBP, at least one socioeconomic/psychosocial factor, and all adjustment variables.
[b] Maximum number of exams that blood pressure was measured for each participant.
