# Supplementary material for: Gene-by-Psychosocial Factor Interactions Influence Diastolic Blood Pressure in European and African Ancestry Populations: Meta-Analysis of Four Cohort Studies
Source: Int J Environ Res Public Health. 2017 Dec 18;14(12):1596. doi: 10.3390/ijerph14121596 (PMC5751013; doi:10.3390/ijerph14121596)
Supplement: Supplementary file 1 [file ijerph-14-01596-s001.zip › Smith IJERPH 10_01_2017 SuppTable2.pdf]

Supplemental Table 2: Demographic, blood pressure, socioeconomic, and psychosocial measures for each cohort

Variable	ARIC				HRS [a]		JHS			MESA			
	Exam 1	Exam 2	Exam 3	Exam 4	Exam 1	Exam 2	Exam 1	Exam 2	Exam 3	Exam 1	Exam 2	Exam 3	Exam 4
**Sex**	x	o	o	o	x	o	x	o	o	x	o	o	o
**Age**	x	x	x	x	x	x	x	x	x	x	x	x	x
**Body Mass Index**	x	x	x	x	x	x	x	x	x	x	x	x	x
**Systolic BP**	x	x	x	x	x	x	x	x	x	x	x	x	x
**Diastolic BP**	x	x	x	x	x	x	x	x	x	x	x	x	x
**Adult Education**	x	o	o	o	x	o	x	o	o	x	o	o	o
**Parental Education**	o	o	o	x	x	o	x	o	o	o	x	o	o
**Anger**	-	x	o	x	x	x	x	o	x	x	o	x	o
**Depressive Symptoms**	-	x	o	o	x	x	x	o	o	x	o	x	o
**Chronic Burden**	-	-	-	-	x/-[b]	x	-	-	x	x	o	x	o

x = exam that the variable was measured; o = exam that the variable was imputed (carried forward or, in the case of parental education, backward from a previous measure); dash (-) indicates that the variable was not measured or imputed for that exam.

[a] Blood pressure was measured at alternating exam dates (during the face-to-face exam only) for HRS participants. Exam 1 indicates the first exam at which blood pressure was measured (2006, 2008, or 2010).

[b] Number of chronic burdens was not measured in HRS in 2008.
