# Supplementary material for: Gene-by-Psychosocial Factor Interactions Influence Diastolic Blood Pressure in European and African Ancestry Populations: Meta-Analysis of Four Cohort Studies
Source: Int J Environ Res Public Health. 2017 Dec 18;14(12):1596. doi: 10.3390/ijerph14121596 (PMC5751013; doi:10.3390/ijerph14121596)
Supplement: Supplementary file 1 [file ijerph-14-01596-s001.zip › Smith IJERPH 10_01_2017 SuppTable3.pdf]

Supplemental Table 3: Genomic regions selected for analysis

Genomic region	GWAS Study	Chromosome	Start Position[a]	Stop Position[a]
ARHGAP42	ICBP, 2011 [1]	11	100553384	100867668
ATP2B1	ICBP, 2011 [1]	12	89976828	90108077
BAG6	ICBP, 2011 [1]	6	31606805	31620953
C10orf107	ICBP, 2011 [1]	10	63417719	63531524
CACNB2	ICBP, 2011 [1]	10	18424606	18835798
CSK	ICBP, 2011 [1]	15	75074425	75095539
FES	ICBP, 2011 [1]	15	91421925	91444006
GOSR2	ICBP, 2011 [1]	17	44995483	45110003
GUCY1A3	ICBP, 2011 [1]	4	156582863	156658501
HFE	ICBP, 2011 [1]	6	26082509	26103571
MECOM	ICBP, 2011 [1]	3	168796287	169386406
MTHFR	ICBP, 2011 [1]	1	11840780	11871977
NT5C2	ICBP, 2011 [1]	10	104840940	104958056
PLCE1	ICBP, 2011 [1]	10	95748746	96097580
PLEKHA7	Franceschini, 2013 [2]	11	16794842	17040990
PLEKHG1	ICBP, 2011 [1]	6	150915999	151169799
rs10850411	ICBP, 2011 [1]	12	115337796	115437796
rs1173771	ICBP, 2011 [1]	5	32765028	32865028
rs11953630	ICBP, 2011 [1]	5	157795402	157895402
rs13082711	ICBP, 2011 [1]	3	27487909	27587909
rs13209747	Franceschini, 2013 [2]	6	127065454	127165454
rs1327235	ICBP, 2011 [1]	20	10919030	11019030
rs1458038	ICBP, 2011 [1]	4	81114723	81214723
rs17428471	Franceschini, 2013 [2]	7	27287867	27387867
rs2932538	ICBP, 2011 [1]	1	113196543	113296543
rs4373814	ICBP, 2011 [1]	10	18369972	18469972
rs7129220	ICBP, 2011 [1]	11	10300538	10400538
SH2B3	ICBP, 2011 [1]	12	111838752	111894427
SLC39A8	ICBP, 2011 [1]	4	103167198	103357415
SOX6	Franceschini, 2013 [2]	11	15982995	16766138
ULK4	ICBP, 2011 [1]; Franceschini, 2013 [2]	3	41283090	42008922
ZNF652	ICBP, 2011 [1]	17	47361568	47444835
ZNF831	ICBP, 2011 [1]	20	57735895	57834168

[a] Start and stop positions for the genomic region include buffers (+/-5kb for gene regions; +/-50kb for non-gene regions).

References

1. International Consortium for Blood Pressure Genome-Wide Association Studies; Ehret, G.B.; Munroe, P.B.; Rice, K.M.; Bochud, M.; Johnson, A.D.; Chasman, D.I.; Smith, A.V.; Tobin, M.D.; Verwoert, G.C. *et al.* Genetic Variants in Novel Pathways Influence Blood Pressure and Cardiovascular Disease Risk. *Nature* **2011**, *478*, 103-109.
2. Franceschini, N.; Fox, E.; Zhang, Z.; Edwards, T.L.; Nalls, M.A.; Sung, Y.J.; Tayo, B.O.; Sun, Y.V.; Gottesman, O.; Adeyemo, A. *et al.* Genome-Wide Association Analysis of Blood-Pressure Traits in African-Ancestry Individuals Reveals Common Associated Genes in African and Non-African Populations. *Am. J. Hum. Genet.* **2013**, *93*, 545-554.
